# Defining motility in the *Staphylococci*

**DOI:** 10.1007/s00018-017-2507-z

**Published:** 2017-04-04

**Authors:** Eric J. G. Pollitt, Stephen P. Diggle

**Affiliations:** 1 Department of Biomedical Science, Western Bank, University of Sheffield, Sheffield, UK; 2 School of Biological Sciences, Georgia Institute of Technology, Atlanta, GA USA

**Keywords:** *Staphylococcus aureus*, Motility, Gliding, Quorum sensing

## Abstract

The ability of bacteria to move is critical for their survival in diverse environments and multiple ways have evolved to achieve this. Two forms of motility have recently been described for *Staphylococcus aureus*, an organism previously considered to be non-motile. One form is called spreading, which is a type of sliding motility and the second form involves comet formation, which has many observable characteristics associated with gliding motility. Darting motility has also been observed in *Staphylococcus epidermidis*. This review describes how motility is defined and how we distinguish between passive and active motility. We discuss the characteristics of the various forms of *Staphylococci* motility, the molecular mechanisms involved and the potential future research directions.

## Introduction

Bacteria are able to move and colonise surfaces in a number of different ways. Bacterial movement can either be active, using energy-dependent cellular mechanisms whereby the bacteria can directly control where they move, or passive, relying on modification of the environment to generate forces that propel the cells. Bacterial motility is important because it is often an adaptation required for survival and dissemination. Being mobile has been linked to colonisation of many different types of surfaces and hosts, antibiotic resistance and coordinated group behaviours and is also known to be important for virulence in a number of pathogenic bacterial species [1, 2]. This has made motility an important area of research focus aimed at elucidating its role in virulence and a possible target for vaccines [3, 4].

*Staphylococcus aureus* is a major human pathogen which is known for its ability to cause a diverse set of infections ranging from superficial skin infections, to life-threatening infections such as osteomyelitis and infective endocarditis. It is a serious clinical problem as it readily and persistently colonises humans (around a third of the population). Antibiotic resistance has become common (e.g. Methicillin Resistant *S. aureus*, MRSA) and no effective vaccine has been developed [5, 6]. *S. aureus* has historically been regarded as non-motile, but recently it has been shown to move over soft agar in two ways: by spreading and by comet formation [7, 8]. It has also been shown that *Staphylococcus epidermidis* and *Staphylococcus xylosus* can spread over soft agar surfaces [7, 9]. There have been some investigations into the molecular mechanisms underlying *Staphylococcus* spreading motility. For example, it has been shown that spreading is closely associated with the *agr* quorum sensing (QS) system and the production of the *agr-*dependent phenol soluble modulins (PSMs), which act as surfactants [10–13]. Here we discuss how motility is defined, how it is relevant to the behaviours observed in the *Staphylococci* and examine in detail the two forms of motility associated with *S. aureus*: spreading and comet formation. We also describe the darting motility seen in some Staphylococcal species and discuss how these forms of motility differ, the molecular mechanisms associated with each of them and consider future directions for the research field.

## Defining active and passive motility

Key to the study of any motility mechanism is to determine whether it is active or passive. Active motility requires an energy-dependent mechanism whereby the bacteria can direct where they go. Passive motility is dependent on modulating the surroundings to generate movement. Active motility is broadly distinguished from passive motility in two ways: (1) by demonstrating previously defined characteristics that are only associated with a known form of motility (e.g. observation of flagella on a bacterial cell suggests it may be capable of swimming motility) and/or (2) identifying situations where the movement displayed can only be explained by active motility (e.g. the observation of the particular running movement of swimming bacteria cannot be explained by passive forces) [14]. Henrichsen carried out the seminal work on defining motility in his paper “Bacterial surface translocation: a survey and a classification”. He performed a survey of motile bacteria and analysed how they moved across surfaces and the characteristics which defined the various types of observed movement [14]. Six types of motility were identified and formally defined. Four were active, inherently requiring a molecular mechanism of propulsion. These were swimming, swarming, gliding and twitching. These forms of motility were actually defined before the underlying molecular mechanisms were discovered and indeed have been highly useful in defining what phenotypes to look for. From these phenotypes, the discovery of mutants not demonstrating the motility phenotype led to the development of the models of the molecular mechanisms underlying the various forms of motility. With gliding motility in particular, the mechanistic basis in many bacterial species remains unknown [15]. Two forms of movement were described as passive, where the motive force is generated by the bacterial community modifying the environment, resulting in movement; these were sliding and darting. The definitions of the types of motility and the basis for each of them are summarised in Table 1.

**Table 1:** The different types of bacterial motility and distinguishing features

Type of motility	Example species	Original description in Henrichsen [14]	Molecular mechanism	Other associated factors	Comments
Swimming	*E. coli, P. aeruginosa*	“The micromorphological pattern is unorganised. The cells move individually and at random in the same manner as flagellated bacteria in wet mounts”	Flagella almost universally required and well conserved in bacteria Bacteria move individually [14]	Chemotaxis is a requirement for swimming and results in the running and tumbling movement	There is one bacterial species that swims without flagella [15]. Archaea use a modified type IV pili as their flagella, it is sometimes called the Archaellum
Swarming	*P. mirabilis* *P. aeruginosa* *B. subtilis*	“Swarming is a kind of surface translocation produced through the action of flagella but (it) is different from swimming. The movement is continuous and regularly follows the long axis of the cells, which are predominantly aggregated in bundles during the movement”	Hyperflagellation, cell elongation (both not always present) Bacteria largely move as groups [16]	Slime production, Surfactant production, Quorum sensing (control of surfactant production) Can be chemotaxis independent	There is an historic tendency to call other forms of motility swarming (e.g. *M. xanthus* swarms are actually engaging in gliding motility)
Twitching	*Neisseria, P. aeruginosa*	“Cells move predominantly singly although smaller moving aggregates occur. The movement appears as intermittent and jerky and do not regularly follow the long axis of the cell”	Type IV pili attachment and retraction are responsible for movement [93]	Pili extend and retract to slingshot the bacteria across a surface	Twitching was discovered before type IV pili were identified and it was a long period before retraction was observed[14, 93]
Gliding	*M. xanthus, Beggiatoa, Cyanobacteria, Mycoplasma* *S. aureus* comets	“The movement is continuous and regularly follows the long axis of cells which are predominantly aggregated in bundles”	Very diverse: type IV pili, focal adhesion complexes, slime guns, deformation of outer membrane. Move singly, groups, filaments etc [2, 15, 72–74]	Slime production, Track formation, Gliding and Flagella are mutually exclusive. Pattern of movement dependent on species	Has evolved independently repeatedly; there is still debate about the actual mechanisms/regulation used in multiple species. Multiple mechanisms could be used at the same time
Sliding	*Streptococcus* spp. *S.aureus* spreading	“Sliding is a kind of surface translocation produced by the expansive forces in a growing culture in combination with special surface properties. The micromorphological pattern is that of a uniform sheet of closely packed cells In a single layer. The sheet moves slowly as a unit”	Bacterial growth and the production of surfactants Bacteria only expand en masse from a central colony [7, 14, 19]	The surfactants can be either free or incorporated into the surface of the cells	As argued in the paper, the definition should be broader to also cover *S. aureus* spreading
Darting	*Staphylococcus epidermidis*	“Darting is a kind of surface translocation Produced by the expansive forces developed in an aggregate of cells inside a common capsule and resulting in the ejection of cells from the aggregate.”	Growth periodically overcomes the adhesive forces ejecting cells [14]	Poorly studied; many basic features are unknown (e.g. it is adhesive or electrostatic factors holding the cells together)	It has only been noted in *S. epidermidis* so far (and may have been seen in *S. xylosus*) [9, 14]

A table outlining the feature as associated with motility both historically and the molecular basis/hypothetical explanations for it

Sliding, darting and gliding are all relevant to the discussion of motility in the *Staphylococci*. To be consistent with the historical definitions of motility, spreading and darting are forms of passive motility, but comets resemble gliding and could, therefore, be considered active. All other forms of movement can be excluded, as *Staphylococci* lack the required flagella and type IV pili. Using time-lapse or video microscopy to observe moving bacteria is important in defining motility because it establishes the phenotype and the category of motility. From this, non-motile mutants can then be identified and in turn the mechanism of movement can be determined. Sometimes the types of motility can be confused when not observed closely [16–18].

## Spreading motility in *S. aureus*

### Spreading and sliding

**Fig. 1:**
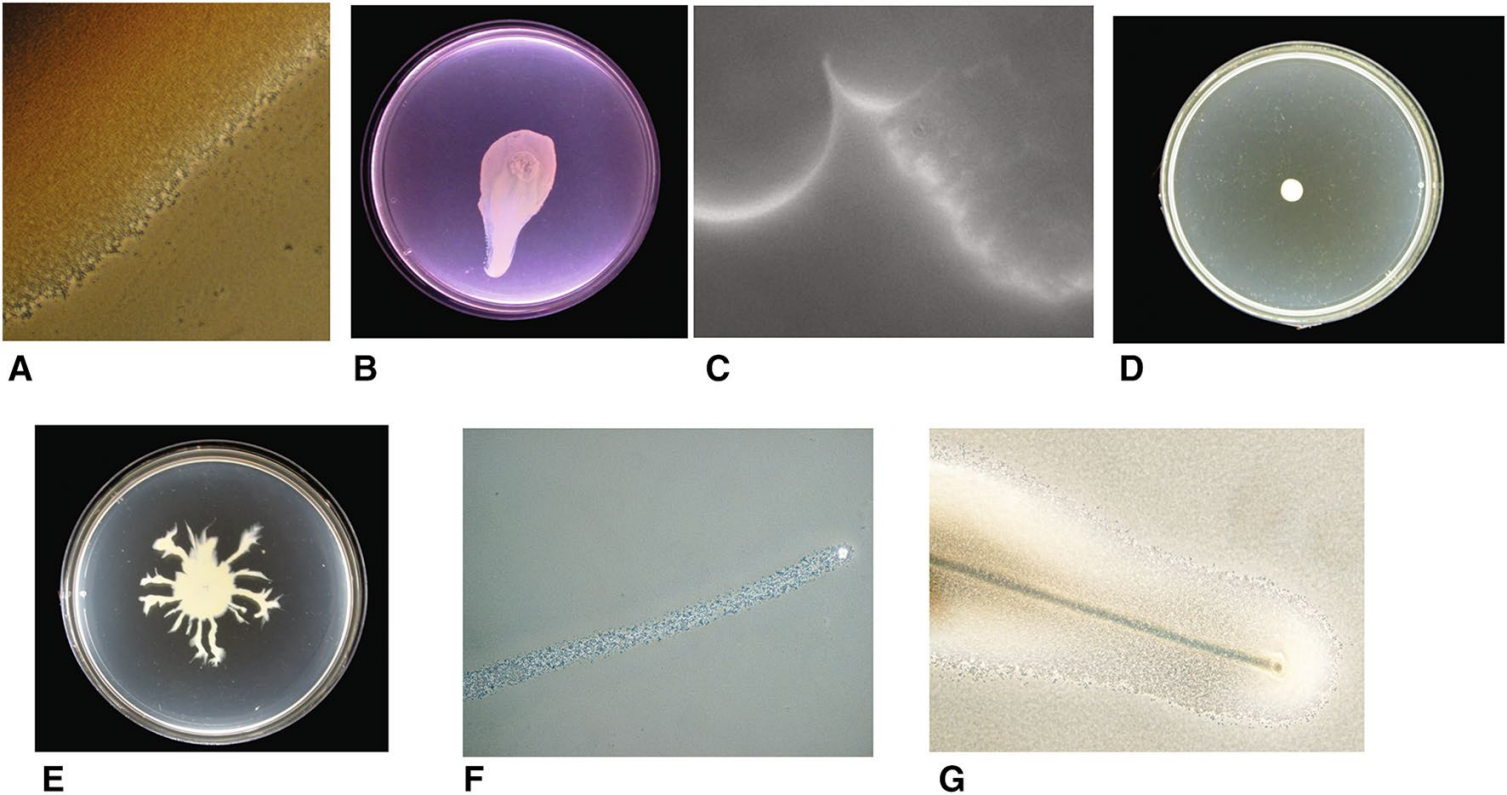
Overview of Staphylococcus motility. **a** The edge of a locally passively spreading colony; **b** a passively spreading SH1000 Staphylococcus colony SH1000; **c** the edge of the surfactant ring that surrounds a motile *S. aureus* colony (spreading from the *top* of the image to the *bottom*); **d** Δ*agr* mutant of the Newman strain showing no spread from its inoculation site; **e** Newman strain of *S. aureus* showing dendrite formation around a central spreading colony; **f** a “comet”: a slime covered aggregate of cells that precede observable dendrites; **g** comets etching the media leaving a track behind them

The first work showing that *S. aureus* can move was described by Kaito et al. [7]. They observed that on motility plates with a low agar concentration, *S. aureus* can spread radially outwards from an inoculation site, forming multiple layers of densely packed cells (Fig. 1a) [7, 19]. This ‘spreading motility’ was interpreted as being most similar to sliding motility but with some appreciably different attributes (Table 1). Spreading results in broadly circular colonies or colonies with large broad lobes extending radially (Fig. 1b). Sliding is a passive behaviour where bacteria are able to move radially outwards, once spotted on soft agar, using growth and surfactant production alone [14, 20]. The growth of bacteria within the colony pushes the bacteria outwards, while surfactant production prevents the cells in the colony from sticking to the surface and each other. A similar effect occurs in *Bacillus subtilis* pellicles, which can climb the walls of glass vessels [21]. Henrichsen stated that sliding bacteria form a monolayer of cells, and bacteria can be observed being pushed out of this layer (by growth) and then falling back into it, pushing the layer of cells outwards. Spreading differs from this in that there are multiple disorganised layers. Research into spreading motility has focused on core *S. aureus* strains of interest such as Newman, SH1000 and USA300, but it has been found that *S. epidermidis* can also move by spreading [7].

Subsequently it was shown that spreading colonies are surrounded by large amounts of surfactant which are essential for movement [11]. A surfactant (surface active agent) is defined as a compound that reduces the surface tension between two liquids or a liquid and a solid. A surfactant can also act as a detergent (disrupting cell membranes), as a wetting agent or as an emulsifying agent depending on its concentration [22]. The surfactant produced by *S. aureus* strains can be observed as a ring around an expanding colony (see Fig. 1c) and once the colony stops moving it dissipates over time. This ring has been shown to be surfactant using the classical drop collapse test [22]. The surfactant also inherently works to encourage the incorporation of water, thus expanding the colony. Time-lapse videos have shown a surfactant ring and that in the early stages of colony formation bacterial cells are carried outwards as a suspension in the surfactant and cells are dropped when the surfactant flowing forwards lacks sufficient force to carry the bacteria forwards [8]. Bacteria can be carried by fluid in the same way sufficiently light particles are. Other time-lapse videos show that there is a further stage where the bacteria form multiple dense layers and large aggregates of cells are pushed forwards by the mass of the colony behind (some individual cells are still moving as if suspended in the surfactant) [19]. Soon after this stage, the colony stops expanding.

**Fig. 2:**
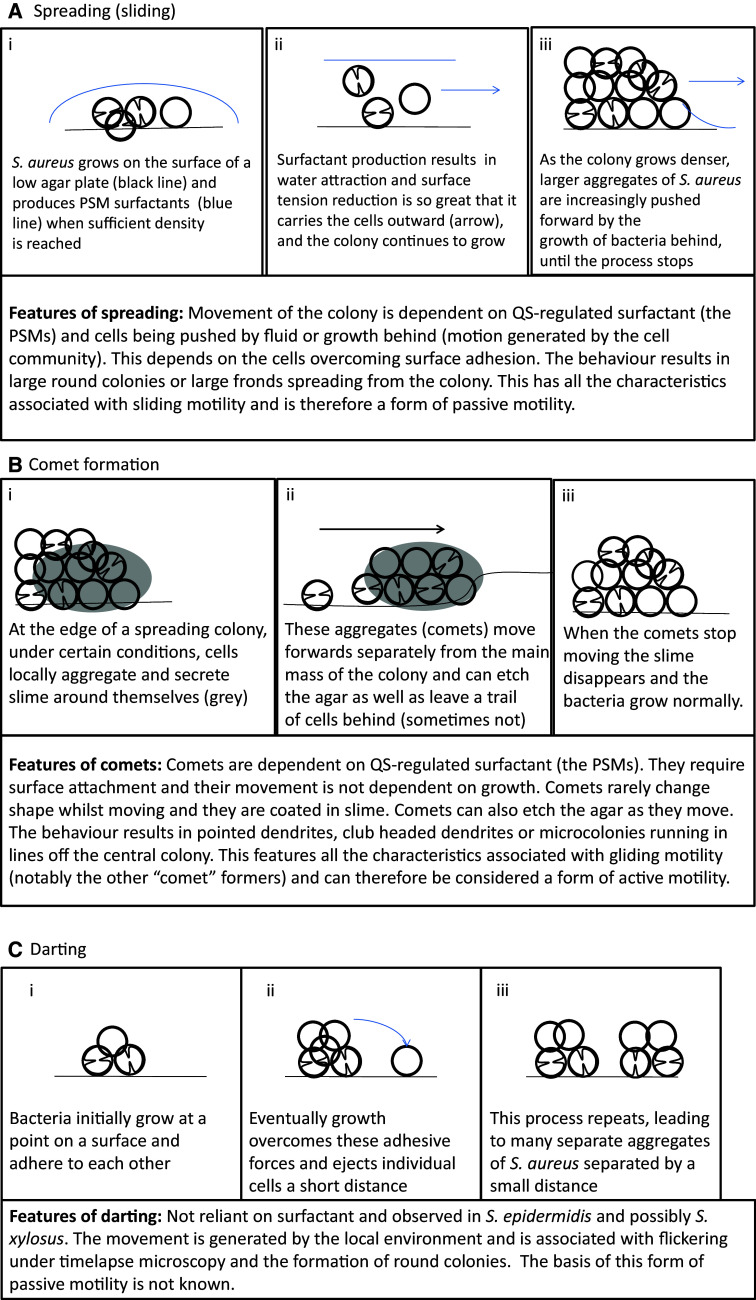
The different forms of Staphylococcus motility. **a** Spreading motility which is a variant of sliding motility has been observed in both *S. aureus* and *S. epidermidis*. (*i*) On the surface of an agar plate the bacteria grow and produce PSM surfactants when sufficient density is reached. (*ii*) The surfactant production, water attraction and reduction in surface tension are so great that it can carry the bacteria outwards, the staphylococci also continue to grow. (*iii*) Finally the growth of bacteria catches up with surfactant production and the bacteria are physically pushed outwards by the colony growth behind, this continues until the colony stops expanding. **b** Comet formation, which has been shown in *S. aureus* and is similar to gliding motility. At a certain point, (*i*) slime covered aggregates of cells form. (*ii*) These emerge from the central colony and can seed cells behind them leading to pointed dendrite formation. These comets interact tightly with the agar and can leave tracks behind. (*iii*) Once the comets stop moving, the slime dissipates and the cells grow outwards. **c** Darting motility which has been demonstrated in *S. epidermidis* and possibly *S. xylosus*. (*i*) Bacteria initially grow at one point. (*ii*) Eventually growth overcomes the adhesive forces holding the microcolony together, ejecting cells a short distance. (*iii*) The process repeats as the colony expands

In light of these findings, we can revisit the initial observation that “spreading is similar to but distinct from sliding” [7]. Spreading has certain characteristics of sliding in that (1) surfactant is important and (2) growth is important for moving the bacterial cells outwards. Initially with spreading, the production of surfactant is so great that it is capable of scattering the individual bacteria and overcoming the attachment of the growing bacteria to a surface (Fig. 2a). As the spreading colony matures, it forms multiple layers where the bacteria are in close contact with each other and can physically push each other forward until they are unable to move further (Fig. 1a). There appears to be a continuum of sliding motility, between where surfactant and the moisture it pulls in dominates as the motive force moving individual cells and where physical pushing of large aggregates of bacteria by the growth of the colony behind is the dominant force. It has been claimed that *Staphylococci* float in the surfactant and the moisture which it attracts; however, other work shows that they are suspended throughout the solution during spreading [8, 19]. The *Staphylococci* also lack the gas vesicles typically required for buoyancy in bacteria [23, 24].

Reviewing the relevant literature, the only difference spreading has from sliding is that it does not form a monolayer of cells [7, 14]. *S. aureus* spreading could, therefore, be a form of sliding if the definition of sliding is expanded to include the formation of multiple layers. It could be the case that *S. aureus* achieves this particular effect through secreting exceptionally large amounts of surfactant. The surfactants involved, the Phenol-Soluble Modulins (PSMs), make up a notably large proportion of what *S. aureus* secretes [25]. We believe that to avoid confusion over classification, that sliding should be more broadly defined as a passive form of motility where (1) surfactant and growth are the main forces driving the movement of bacteria over a surface; and (2) bacterial cells are pushed out by the central mass of the colony.

### Mechanisms associated with *S. aureus* spreading motility

Research on *S. aureus* spreading has focused on the role of the *agr* quorum sensing (QS) locus and the PSMs. Research has also investigated genes that have previously been associated with virulence and biofilm formation. However, because spreading motility is passive, it remains poorly studied and it is difficult to show how relevant it is to natural environments because there is no active molecular motor involved. Motility is generally hard to observe in a natural environment (only swimming motility is readily observable in situ) and the factors involved may have other biological roles [1, 2, 26].

### The *agr* system

**Fig. 3:**
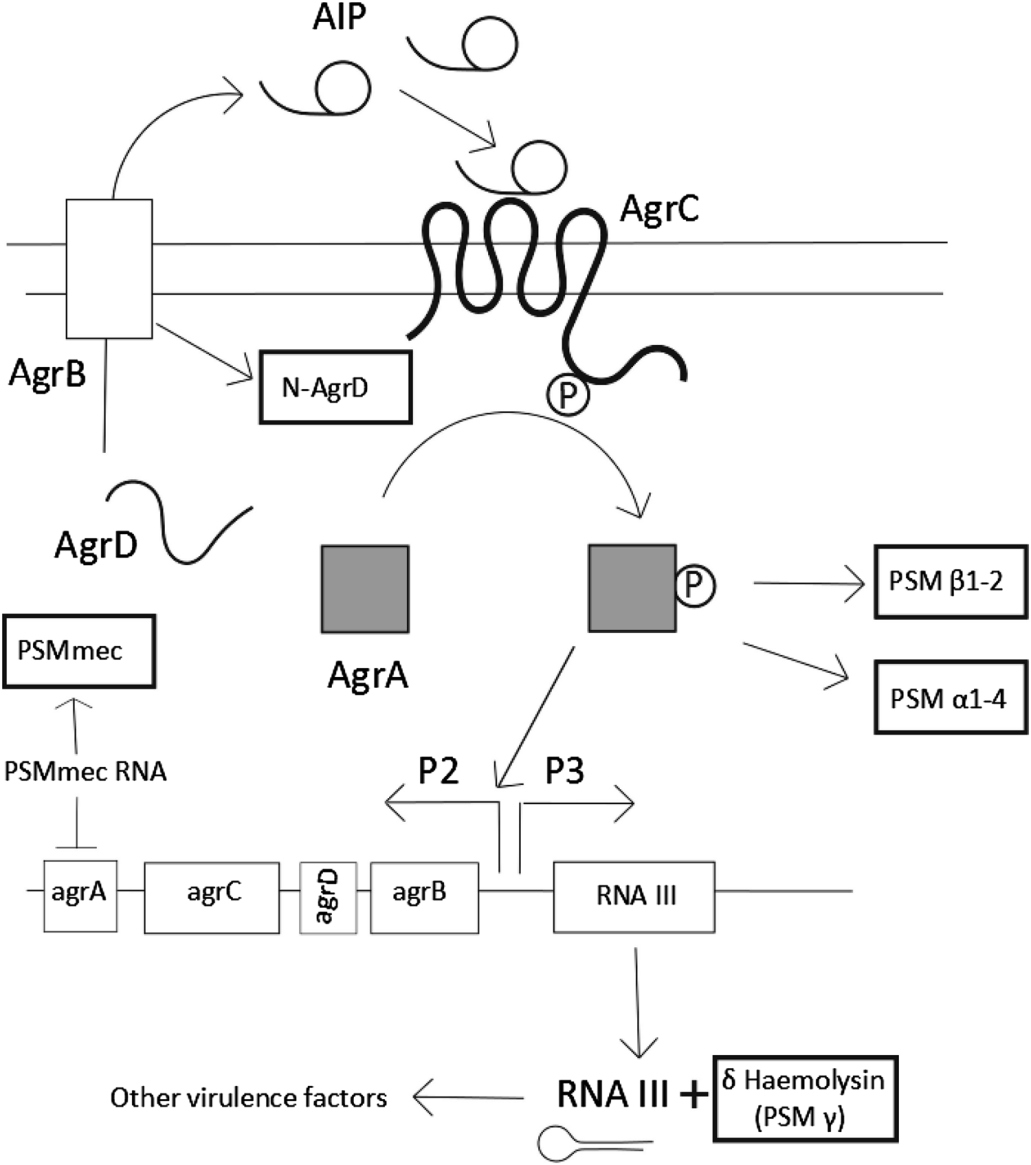
The regulation of the phenol soluble modulins. The PSMs are closely associated with the *agr* quorum sensing system. The different PSMs are highlighted in the *outlined boxes*. The *agr* system controls its own expression through the production of its own signal and receptor. The *agr* system is initially expressed at a low level. At sufficient concentrations of AIP (usually associated with increased cell density) it interacts with the AgrC, which in turn activates AgrA, the response regulator. This then greatly increases expression of *agr* (forming a feedback loop) and also induces the expression of *agr*-dependent products. AgrA can directly upregulate the PSMs whereas most other virulence factors are controlled through the RNAIII internal signal molecule, which also encodes PSM γ (δ-haemolysin)

Several research groups have shown that spreading motility is *agr* dependent, with *agr* mutants being unable to spread from their inoculation site (see Fig. 1d compared to b, e) [11, 12]. *Agr* QS systems represent the archetypal form of QS via peptide signalling in Gram-positive organisms, and was first described in *S. aureus* [27]. QS is a mechanism by which bacteria can detect a threshold density of related bacterial cells due to production and sensing of diffusible signal molecules in the environment. This enables the whole population to make a coordinated response [28–31]. *S. aureus* QS is mediated solely through the *agr* locus (see Fig. 3) which contains the genes *agrABCD*. These encode the signal peptide (AgrD), a signal exporter membrane protein (AgrB), a signal response protein (AgrC) and a response regulator protein (AgrA). The AgrD peptide is truncated and converted by AgrB into its active form, the autoinducer peptide (AIP) [32]. AIPs are signals and only exist outside the cell (AgrB also exports the AIP). In *S. aureus*, the global virulence effects of the *agr* locus are mainly mediated through AgrA-controlled RNAIII (a large RNA that acts as an internal secondary messenger). Other *Staphylococci* have homologs of RNAIII, but only in *S. aureus* has it been linked to the control of a wide variety of virulence factors [33]. Broadly, RNAIII up-regulates the expression of toxins such as α-haemolysin and down-regulates the expression of surface adhesins such as protein A. RNAIII also directly encodes δ-Haemolysin (PSMγ). *agrA* regulates the expression of a small number of proteins, in particular the PSMs (Fig. 3). The remnant of AgrD created during the formation of AIP also has PSM-like properties. The PSMs are the main surfactant involved in spreading and *agr* regulation has not been directly linked to spreading motility by any other mechanism. *Agr* is not initially expressed when *S. aureus* is spotted on a motility plate but its expression is induced after several hours (after the density of bacteria increases) and this correlates with the start of colony expansion and surfactant production [19].

Additionally, different *S. aureus* strains can produce 1 of 4 different AIP types (I–IV). These AIPs have different cognate AgrB and AgrC proteins which can inhibit *agr* systems with different AIP types [34]. It is believed that they represent incipient speciation in *S. aureus* and certain AIP types tend to be found with certain conditions and virulence factors [27]. It has been shown that motility is not dependent on the *agr* type [8] and the effect of *agr* competition/inhibition has not yet been reported in the spreading phenotype.

An interesting point to note regarding *agr* and spreading is with strain RN4220. This is a restriction-deficient strain that is used as an intermediate for cloning but is sometimes used for phenotypic studies, including spreading motility. It has a large number of mutations in addition to ones which affect the *agr* system. In particular, *agrA* has been altered by a slipped mispairing mutation [35]. The consequent reduced activity of *agrA* results in delayed or no RNAIII expression in vitro [35, 36]. Some research groups have reported RN4220 colonies spreading whilst others have reported no spreading, so RN4220 spreading may have been affected by this unusual *agr* mutation [7, 10]. We have observed that movement or no movement occurs randomly under our experimental conditions. Therefore, all *S. aureus* research that uses RN4220 to test motility should be treated with caution and its use for these types of studies is not recommended [36].

### The phenol-soluble modulins (PSMs)

Tsompanidou et al. found that the main surfactants involved in spreading motility are the phenol-soluble modulins (PSMs), which were already known as major *S. aureus* virulence factors. The PSMs mediate a range of behaviours, notably surface colonisation, biofilm maturation, phagosome escape, general pathogenicity, and some also have antimicrobial properties [37]. There are several reviews on the PSMs so this one will focus on their effect on motility, which has received less attention [11, 25, 38]. Most of the behaviours of the PSMs are linked to their powerful surfactant properties. *S. aureus* possesses a variety of PSMs: PSMα1–4, PSMβ1–2 and PSMγ more commonly called δ-haemolysin but PSMs also exist in the other *Staphylococci* [39]. The PSMs discovered so far fall into two groups: the α type peptides (PSMαs and including PSMγ) which are 20–25 amino acids in length, and the β type peptides (PSMβs) which are around 44 amino acids in length [38]. Although they all share a common α-helical structure, they vary in their overall charge and apparent role. PSMs are exported outside the cell by the dedicated ABC transporter, Pmt [40, 41]. PSMs can be secreted in both formylated (f-Methionine) and non-formylated forms and this is apparently dependent on growth conditions [42]. PSM gene expression is regulated via *agr*, often directly by *agrA* (Fig. 3). The regulation of PSMs may represent the original function of *agr* in the *Staphylococci* because they are broadly conserved between species [43]. The many other virulence factors that *S. aureus* possesses are speculated to have evolved later, since they are largely not present in other *Staphylococcus* species [44].

PSMs are the major surfactants involved in spreading. This has been demonstrated both through the exogenous addition of synthetic surfactant as well as the generation of PSM mutants [11, 13, 45]. It was first shown that disruption of the PSMα locus abolished spreading [13]. It was subsequently found, through the addition of synthetic peptides, that the major PSMs responsible for enabling spreading are PSMα3 and PSMγ (δ-haemolysin) [11]. PSM knockout mutants also show reduced spreading, the more PSMs are knocked out, the more spreading is reduced. The PSMα1–4 mutant spreading ability was more reduced (but not completely abolished as seen previously) than that of the PSMβ1–2 mutant. However, the PSMαβ mutant was still able to spread compared with an *agr* mutant (where no spreading was observed) indicating that δ-haemolysin (PSMγ) is likely to account for the remainder. As the same PSMs (PSM α3 and PSMγ) that are important for spreading are also important for virulence, it is likely that the powerful surfactant properties are important for both behaviours [46]. Whereas there is consensus that at least some of the PSMs are critical for spreading motility there is controversy regarding the importance and effect of individual ones. In particular, there is controversy regarding the role of δ-haemolysin (PSMγ) in spreading. Some researchers claim that it has a positive effect on spreading whilst others claim that it has a detrimental effect [11, 47]. It has been demonstrated that the factor in spent supernatant which inhibited spreading was δ-haemolysin [47]. It has also been reported by others that δ-haemolysin expression reduces spreading [48]. On the other hand, Wright et al. have shown that δ-haemolysin is necessary for spreading under similar conditions [49]. We believe it is most parsimonious that δ haemolysin has a positive effect on spreading but unusual factors may be present; δ-haemolysin would not be the first motility self-inhibiting exoproduct to have been identified. It has been shown previously that precursor 3-(3-hydroxyalkanoyloxy) alkanoic acids (HAAs) produced by *Pseudomonas aeruginosa* under the control of the *rhl* QS system inhibit swarming motility, whereas the terminal product rhamnolipids are the surfactant responsible for swarming [50]. This has led to confusion, as they are so chemically similar that initially they were inadvertently co-purified leading to the belief that rhamnolipids were inhibitory [50, 51]. A similar occurrence could be happening in *S. aureus*, as varying mixtures of formylated and non formylated PSMs are known to be secreted depending on growth conditions [42, 52]. The discrepancy between the results could be accounted for by the different mixtures of chemical inhibitor/facilitator. There could also be other factors occurring such as interactions of PSMs at different levels with the bacterial cell membrane and interactions of the PSMs with their own gene regulators [41, 45].

PSMs are also linked to biofilm development but this effect is context specific and appears to be related to the dissemination and structure of the biofilm. *S. aureus* strains which are defective for *agr* or PSMs make denser, smoother and less structured biofilms but can disseminate less easily in an in vivo biofilm model [53, 54]. βPSMs are known to contribute to biofilm maturation. It has also been shown under certain conditions that the PSMs can form amyloids within biofilms and that instead of dispersing biofilms these PSMs can increase biofilm cohesion in a similar manner to the way amyloids can aid biofilm formation in different bacterial species [53]. Interestingly, surfactants that are important for motility in other organisms are also important for biofilm maturation, e.g. *P. aeruginosa* rhamnolipid-deficient biofilms are less structured in the same manner, and rhamnolipids are critical for swarming motility over surfaces [55]. More generally, factors that affect motility in other bacterial species often affect biofilm formation.

### The other PSMs

Other PSM-like proteins have been identified outside the core set of PSMs. Two in particular are PSM-mec and N-AgrD [13, 56]. There may indeed be many more types of PSMs as a mass spectrometry study of USA300 has found many different previously unknown homologues [39, 57]. N-AgrD is the remnant from the AgrD peptide once it has been converted into AIP; it has been recently found to have PSM-like properties, but its effect on spreading has not been investigated [56]. PSM-mec (αPSM group) has been discovered which is not part of the core *S. aureus* genome but instead is found on variants of mobile genetic element, SCCmec, that carries mecA [58]. PSM-mec is, therefore, unique as it is the only known toxin found on a staphylococcal antibiotic resistance cassette. There is some discrepancy in the interpretation of its role in gene regulation which has a follow-on effect on spreading motility. It has been stated that PSM-mec is both *agr* regulated but also encodes an RNA that down-regulates *agr* expression [47, 58–60]. This type of regulatory behaviour is not unique as it has been found that Surfactin (a surfactant required for swarming motility) has a similar effect on *B. subtilis* quorum sensing [61]. Regulatory feedback of PSMs and associated products may also explain the varying effects seen with δ-haemolysin [41]. PSM-mec itself appears to have a minor role in spreading; it enables some spreading of an *agr* mutant but not to the same extent as the other main PSMs implicated in spreading. It has also been shown to neither inhibit nor boost spreading of a WT strain [11]. However, as with δ-haemolysin, there are reports that both PSM-mec and the associated RNA can suppress spreading, the RNA through its interaction with *agr* [60]. It remains to be determined what the dominant effect of the PSM-mec is on spreading, and part of the variation could be due to the strain background and conditions under which it is expressed [58].

### The cell wall and spreading

Conceptually, spreading motility has two major factors governing how well the bacteria move: (1) the surfactant produced, and (2) cell surface factors that govern how well the cells interact with each other and with the surfaces over which they are attempting to spread. The cell surface factors that have been investigated with respect to *S. aureus* spreading include the teichoic acids, lipoteichoic acids and secretory proteins (particularly the sortases) [7, 62]. Teichoic acids form a major component of Gram-positive cell walls and fall into two categories: wall teichoic acids and lipoteichoic acids. Wall teichoic acids are directly bound to the peptidoglycan whilst lipoteichoic acids are anchored to the bacterial surface membrane [63]. The teichoic acids compose 40% of the dry weight of the cell wall but their production and use can be disrupted and *S. aureus* still remains viable *in vitro* despite morphological defects. Kaito et al. disrupted both teichoic acid production and modification through knocking out tagO (disrupts early synthesis), and the *dlt* operon (addition of D-alanine to teichoic acids) and found that both had greatly reduced spreading ability [7]. A *ypfP* knockout also had reduced spreading ability. YpfP alters the glycolipids to which lipoteichoic acids anchor, so they are able to move freely in the membrane and hence are likely to inhibit associated proteins. The importance of the teichoic acids for spreading is further underlined through the identification of the *msrR* gene, which belongs to the LytR-CpsA-Psr family involved in cell division. An *msrR* mutant shows reduced production of teichoic acids and has also been shown to have reduced spreading, indicating that teichoic acids are important for spreading motility [64]. Membrane function has also been examined in a limited way via the *mprF* mutant (required for the synthesis of the phospholipid lysylphosphatidylglycerol) but found not to have an effect on spreading motility [7].

Tsompanidou et al. [62] have looked further at the secretion proteins CidA, DsbA, Lgt, LrgA, IspA, PrsA, SecG, SecY2, SpsA, SrtA, SrtB, TatA, TatC, and MscL. They found that only a *srtA* mutant had an altered spreading phenotype, and unusually spreading was increased. SrtA (sortaseA) is an export protein that exports and maintains proteins at the cell surface which have the following motif LPxTG. It is important for virulence, likely due to the proteins exported to the surface being directly involved in virulence such as protein A. However, the work focused mainly on the related exported proteins involved in adhesion; FnbpA, FnbpB, ClfA and ClfB. They found that knocking out all these genes simultaneously resulted in a significant increase in spreading. So by decreasing adherence, it could be the case that spreading is increased. In other bacteria factors that increase motility can also be correlated with decreased biofilm formation and adherence [65].

### Other factors and spreading

Several other factors have also been investigated for their association with spreading; these include extracellular DNA and *fudoh* [66, 67]. The presence of extracellular DNA has previously been linked to biofilm formation in multiple bacterial species as it acts as a scaffold holding biofilms together; however, in some species it is associated with increased dispersal [68]. It is, therefore, unsurprising that it was found to have an inhibitory role in spreading [66]. Spreading was reduced when secreted nucleases *nuc1* and *nuc2* were deleted. Spreading could be restored through the addition of DNase, and *nuc1* and *nuc2* were shown to be particularly active on the edge of the colony. The direct role of extracellular DNA and its digestion in spreading is unknown, but it is feasible that the centre of a spreading colony behaves like a biofilm where extracellular DNA is important for cohesion and its breaking down leads to dispersal [69]. A putative gene *fudoh* was also identified based on certain SCCmecs lacking spreading ability [67]. However, it has been stated by others that it lacks a Shine–Dalgarno sequence in front of the protein and so should not create a functional protein [70]. It is also very close to the psm-mec gene, so attempts to disrupt *fudoh* may have also disrupted psm-mec leading to an observable spreading phenotype [70].

## Comet formation in *S. aureus*

### Observations of comet formation

*Staphylococcus aureus* also engages in comet formation, which is distinct from spreading behaviour and fits the definition of gliding motility. Pollitt et al. found that observable dendrites can be formed which were preceded by “comets” of motile cells and which have characteristics of gliding motility, a form of active motility (see Fig. 1e, f) [8]. Phase contrast microscopy revealed that at the tips of pointed dendrites, there were phase bright objects which disappeared over time. Subsequent investigations showed that these phase bright objects were groups of cells covered by slime and that they were moving forwards leaving a trail of cells behind them which formed the comets (see Fig. 1f). These trails of cells subsequently formed dendrites. This was observed in a broad range of *S. aureus* strains, covering a range of *agr* types. Under certain conditions the comets were able to etch tracks in the agar which revealed their previous trajectories (see Fig. 1g). The tracks also showed that occasionally comets could move without leaving a continuous trail of cells, explaining why sometimes microcolonies in lines can be observed running off the main colony [71]. The comets have characteristics associated with gliding motility, a form of active motility, and not with any known forms of passive motility (as summarised in Table 1) [14, 72, 73]. The characteristics associated with gliding are (1) discrete comet movement (they are not readily moved by fluid unlike the bacteria in the tails and are not being pushed by the colony mass); (2) resemblance to known gliding ‘comets’ formed by other bacterial species (see below); (3) slime is present around the moving comets; (4) the occurrence of tracks behind the comets (this is particularly associated with gliding); (5) the lack of flagella; (6) contact with the agar surface is required for movement [72]. This behaviour also requires PSM surfactants and the comets move out into the surfactant ring surrounding the colony (Fig. 1c). A model of this movement is proposed in Fig. 2b.

Gliding is a form of active motility found in a wide variety of bacterial species that is not dependent on flagella and is defined by its smooth and linear motion [2, 14, 15, 72–74]. Within this definition there are wide variations; bacteria can move either singly (e.g. *Mycoplasma*) in irregular spontaneous groupings, in well-defined groupings of a consistent type or as filaments, e.g. (*Beggiatoa*) [75, 76]. Some forms of gliding only allow travel continuously in one direction, others move in wheeling circles whilst others can engage in periodic reversals. Gliding has been proposed to work using a number of different mechanisms. These include focal adhesion complexes in the cell membrane, slime nozzles that generate gel-like slime that expand to push the bacteria forwards, type IV pili, and deformation of the membrane by a high–low cargo push system acting against the slime [15, 77, 78]. Some gliding bacteria can have multiple independent motility systems and may use some of the above mechanisms in combination, such as *M. xanthus* [79]. The various forms of gliding movement in the different bacterial species generally have no common evolutionary origin.

The most commonly studied gliding bacteria are *M. xanthus, Mycoplasma, Flavobacterium johnsoniae* and the *Cyanobacteria* [15, 75, 77, 80]. *S. aureus* comet formation fits within the universal definition of gliding, although it has not been shown to be motile as individual bacterial cells, as are the most commonly studied gliding bacteria. There are some species of gliding bacteria that do not move as individual cells [72]. Notably *S. aureus* comet formation resembles motility seen in other previously described gliding bacteria, *Pseudanabaena* and *Isosphaera pallida* [81, 82]. These gliding bacteria form aggregates of cells that were also independently called comets and which move forward as stable groups, seeding cells behind. Comets also have overall similarities to the group motility of *Synechocystis* sp. PCC 6803, a cocci cyanobacteria, which forms finger-like projections led by large aggregates of cells (observationally very like comets) in addition to individual twitching motility (*Synechocystis* has type IV pili but the basis for movement as a group is not known) [83–85].

It was previously investigated whether the comet movement could be explained by the comet being pushed by fluid or other mechanisms that relied on the bacteria not being attached to the surface of the media. This was done by adding a droplet of PBS nearby; it was observed that although the bacteria in the tail could be readily pushed away by the PBS, the comet head was not affected [8]. This indicates that the comet head is attached to the surface of the agar and surface contact is another requirement for gliding motility. This aligns with the observation that large aggregates of bacteria are left behind, and if surfactant was the primary force pushing the comet tip, then the large aggregates should also move as readily as the comet tip. It has been suggested to the authors that the comet head is producing a surfactant gradient and diving forward on it. Interestingly this resembles the earlier tension gradient theory of gliding propulsion and is theoretically plausible (see Table 1) [86].

The observation that *S. aureus* could be actively motile is radical and challenges the long-standing belief that *S. aureus* is non-motile [87]. The previous absence of reported observations could be due to the significant difficultly in detecting many forms of motility unless the critical environmental requirements are realised under experimental conditions (swimming motility is an exception) [14]. It is also unlikely to be observed by chance, as *S. aureus*, like many other gliding bacterial species, lacks observable appendages [2]. It is interesting to note that the observed pointed dendrites also occur intermittently in previously published work on spreading motility, both in certain strains and in certain mutants such as the αPSM mutants [11]. Sharp dendrites were not observed in studies which focused on passive spreading behaviour, although some finger-like projections were seen [19]. These could be investigated further, especially because spreading behaviour theoretically produces round colonies by default [14]. The precise conditions that lead to comet and dendrite formation are currently unknown; they may be a response to the drying of the media, in much the same way that bacteria can switch from swimming to swarming as they expand across drying media [16].

### Molecular mechanisms underlying comet formation

As comet formation has only recently been described, the underlying mechanisms remain to be determined. Comet formation relies on surfactant-like spreading motility to prevent the bacteria adhering to the surface and, therefore, it is likely that the PSMs are one of the factors enabling the comets to move. Comets are only seen within a ring of surfactant surrounding the colony. The other core factors that remain to be determined are the components of the slime and the actual mechanism by which the bacteria move forwards. Also, the cells within comets may be growing differently as it is hard to explain how comets can secrete large numbers of bacteria and yet not apparently change the shape or the overall organisation of the comet aggregate.

The slime around the bacteria in the comets may be difficult to determine given the proportionally low amounts that are produced. *S. aureus* produces slime in a number of other situations and this has been studied most in the context of biofilm formation. Biofilms are either PIA/PNAG dependent or independent [88]. PIA/PNAG-based biofilms are held together by the PIA glycan whereas the PIA/PNAG-independent biofilms are held together by assorted surface adhesins and extracellular DNA [89]. However, none of these compounds appear to completely fit with the slime that is observed around the comets which is dense but has some fibres within it [8]; PIA biofilms are very smooth and globular whilst the PIA-independent biofilms secrete little, if any, slime and are all intended to keep the bacteria stationary on a surface [90, 91]. The PSMs, whilst very fibrous, have not been shown to align themselves as slime, and also amyloid formation is inhibited by TSB (on which the motility assay is based) [53]. The other reason these compounds are unlikely to be responsible for the observed slime is that when they form in biofilms, they are distributed everywhere and adhere the bacteria to a fixed spot, whilst in a motile colony they are only found in mobile comets on the edge of the colony and not in the rest of the motile colony [92]. Hence, based on the currently available information, it seems likely that the comet slime is either a new compound(s) or one of the previously described compounds acting in an unknown manner.

The mechanism of locomotion of the comets also remains unknown. It could be one of several mechanisms associated with gliding motility; focal adhesion complexes, deformation of the membrane by high cargo push against secreted slime, secretion of expandable slime via slime nozzles, the directed manipulation of surfactant tension gradient or some unknown mechanism [15, 72, 77, 78, 86]. It remains to be determined how much contact there is between *S. aureus* and the substrate and whether there is slime between them. It also remains to be determined how the movement is coordinated between the *Staphylococci* in the comet head. Interesting ways to solve the coordination issue are proposed in other bacterial species that move in groups [93, 94]. An interesting hypothesis is that *S. aureus* cells need to aggregate to move effectively. Cocci intrinsically lack polarity (unlike bacilli) and aggregating to form a comet would allow them to orientate themselves, which also may explain why the behaviour is seen in other surface motile cocci [81, 83, 95].

It also remains to be determined to what extent comets are capable of taxis (movement towards or away from a stimulus). Comets are capable of stopping and bending away from other *Staphylococci* colonies and other dendrites from the same colony and this may represent taxis but the mechanism for this is unknown, apart from appearing dependent on the other colony producing surfactant (*agr* mutants are not recognised and are collided with) [8]. *S. aureus* lacks variants of the genes commonly required for chemotaxis, but there is not a complete link with chemotaxis and motility beyond swimming motility [16, 96]. Demonstrating taxis can be challenging outside of chemotaxis in swimming bacteria and the observations of phototaxis [83, 97]. This is largely due to interactions with the physical surface and the bacteria not being completely free to move (particularly when moving in groups), also chemotaxis is not required for some forms of motility in some bacteria [16, 98]; for instance Mycoplasmas are actively motile, yet lack the chemotaxis genes, but some researchers have found they are chemotactic whilst others disagree [99, 100].

## Darting motility in the *Staphylococci*

Darting is a form of passive motility, which has also been intermittently linked to *Staphylococci* [14]. It occurs where the bacteria are believed to form clusters of cells on a surface and then grow. Eventually the growth explosively overcomes the adhesive forces keeping the cluster together (seen as flickering of the growing cells under the microscope). A few cells are then ejected short distances and the process repeats as the colony expands. This results in the surface pattern of aggregates of bacteria separated by narrow regions where the bacteria are absent (a model is presented in Fig. 2c). It has not been well characterised beyond appearing in Henrichsen’s study, and to date it has only been linked to *S. epidermidis* and has not been seen in other genera of bacteria [14]. However, *S. xylosus* has been observed forming large colonies on low-agar plates that appear distinct from spreading and comet formation [9]. In particular, *S. xylosus* lacks surfactant production and the organisation of the bacteria is distinct from spreading or comet formation. The distribution appears very much like that seen in darting (large aggregates of cells are separated by narrow gaps) and both *S. epidermidis* and *S. xylosus* exhibit darting behaviour on low-percentage agar BHI plates [9, 14]. It would require additional microscopic observation over time to investigate whether the characteristic flickering is present otherwise the observations are consistent with darting. If darting is present in both, it would be interesting to see how prevalent this behaviour is in the *Staphylococci* under the same conditions.

## Future directions and research questions

As *Staphylococcus* motility is a relatively new research area, there are many avenues to follow and there remain certain areas that are subject to debate. There are areas specific to *S. aureus* such as defining the requirements for comet formation versus spreading. There are also general themes which have been found to be important regarding motility in other bacterial species such as the relationship between motility and virulence, motility and biofilm formation, motility as a vaccine candidate target and the physical parameters involved in motility.

### Assay development

The metabolic and physical requirements of spreading, darting and comet formation in *S. aureus* need to be investigated further. In particular, as spreading and comet formation occur under similar conditions, a completely defined assay needs to be developed that will enable a single motility behaviour to be isolated. There needs to be a focus on this issue as it has caused problems with studying mutants in other bacterial motility assays, for example, swimming and swarming have been confused in *B. subtilis* [16, 17]. In particular, the large dendrites observed by Li et al. need to be resolved; they are not pointed like the dendrites formed by comets. They could be due to some variation of comets or spreading (which forms large fronds anyway); both gliding and sliding motility have previously been associated with the formation of dendrites (though sliding is generally more associated with round colonies) [14, 101]. Furthermore, defined assays will have the additional benefit of enabling the metabolic requirements for the various types of motility to be studied. This has been done explicitly with *P. aeruginosa* swarming where the variables involved have been examined and efforts have been made to find the best possible assay [102]. Motility assays are useful as simple and quick ways to test the effects of new compounds, generated mutants and novel research topics [16, 103]. The spreading assay has already been used to screen a variety of compounds such as ferulic and gallic acids and isothiocyanates [104, 105]. The effect of blood serum on spreading has also been tested and used to determine which blood components can stimulate spreading in various *S. aureus* isolates [106]. *S. aureus* motility assays could also be used to study social evolution as has been done previously in virulence models and with other bacterial motility assays [71, 107, 108]. In particular, how social behaviours impact quorum-sensing controlled surfactants and motility has been studied in other pathogens so it could be highly relevant to study the same phenomena in *S. aureus* motility and determine if similar dynamics are observed [109, 110].

### The role of PSMs

PSMs are key surfactants in spreading and comet formation, but a number of key questions remain to be answered. For example, why are so many different variants of PSMs produced and why are some formylated and others not? Formylated proteins are a known pathogen-associated molecular pattern (PAMP) and pathogenic bacteria have evolved ways to hide them, hence why does *S. aureus* excrete them in large amounts and why are there so many different variants compared with other bacteria [42, 52]? An important technique that has been used to pursue surfactant production in the context of motility is imaging mass spectrometry [111, 112]. This has been used frequently with *B. subtilis* swarming [111]. It would be interesting to investigate where the various PSMs and other factors are distributed within a motile colony. There is also the further question of how PSMs are aligned and physically arranged when used as surfactants. They are likely to be arranged differently from when certain PSMs are being used to disrupt cell membranes. When sufficient amounts of PSMs insert into membranes, they form pores leading to the breakdown of the membrane, so it is likely their arrangement when aiding motility is going to be rather different [113]. Research is being undertaken into how PSMs are interacting with each other, the bacterial surface, gene regulation and the agar surface [41, 45, 53, 113].

### Cellular changes in spreading

As previously discussed, cell wall components, such as teichoic acids, are important in spreading [7]. However, these factors are important not just for cell structure but also for the adhesion of surface proteins and excretion of cytoplasmic proteins, so it remains to be determined which of these factors is critical for motility [63]. There are potentially other factors worthy of investigation involved in spreading motility beyond simply growth and surfactant production, for example, the importance of cell surface factors in determining growth over the surface and interaction with other cells [114].

### Cellular changes in comet formation

It is likely that different intrinsic cell factors are more important in comet formation than in spreading. In particular, the aspects that need to be investigated are the slime around the comet, how the cells in the comet are growing and the mechanism by which they move. With ordinary spreading, the cells can be seen dividing in the classical manner [8, 115]; however, comets can end up shedding large numbers of cells, yet not change shape or break apart. This raises interesting questions as to how this is physically achieved both in terms of displacing cells and also in terms of cellular changes. Determining the chemical basis of the slime is important but challenging because obtaining sufficient amounts and separating it from the agar is likely to be difficult. It would also be interesting to discover the extent to which comets can move around in response to different physical factors. The motility mechanism could be unravelled using transposon screens [116].

### Virulence and vaccines

Bacterial motility is well known for its importance in virulence and colonisation, and is consequently a target for inhibition and vaccine development. With other bacterial species, the work has focused on type IV pili and flagella [3, 4, 117]. In general, motility in pathogens is important for initial colonisation and the spread of infection. It can be difficult to translate behaviour demonstrated on motility plates into observations in vivo and this is particularly the case with passive motility [16]. For instance, showing that PSMs are needed for colonising chicken tissue does not conclusively demonstrate that spreading is required, as PSMs are also important for the destruction of tissue and this could be the relevant factor in that case [11, 46]. The concept that *S. aureus* can be actively motile is likely to have a significant impact on interpretations of how *S. aureus* initiates infections. Currently *S. aureus* is believed to be introduced into the host through a break in tissue barriers [5]. This is largely due to it being assumed that it is non-motile and, therefore, unable to break through the barrier. If it is motile in tissues, then combining this with its arsenal of virulence factors means that *S. aureus* has all the tools to make its own entry into a host. For instance, *Neisseria meningitidis* uses its motility mechanism (type IV pili) in conjunction with virulence factors to escape the nasal cavity and proceed to cause deep infections such as meningitis and septicaemia. Interestingly, in approximately a third of ‘deep’ *S. aureus* infections, the source of infection cannot be determined and consequently it is believed that the disruption in the tissue barriers is too small to detect [118]. Motility has been used to explain situations where *S. aureus* has entered tissues distal from the site of the original infection where a passive mechanism would be difficult to explain [119]. It would also be interesting to investigate the extent to which various forms of motility are related to biofilm formation because it is generally believed that there is a continuum between sessile (biofilms) and motile behaviours [16, 120].

Vaccine candidates have not been developed for gliding bacteria but could conceptually be developed as active motility is generally critical for pathogenic bacteria particularly in early colonisation [121, 122]. Motility could be a target for novel therapeutics and vaccines and so provide new ways to attack *S. aureus* which is important because of the increased spread of dangerous antibiotic resistant strains. Several factors related to spreading such as *agr* and the PSMs are already being targeted with inhibitors due to being important virulence factors in their own right [123–126].

## Concluding remarks

This review shows that the study of Staphylococcal motility is a dynamic research topic and of interest to multiple groups both in terms of investigating *S. aureus* virulence and *S. aureus* surface colonisation mechanisms. *S. aureus* spreading has now been well defined and depends on the PSM surfactants. Darting motility has also been previously observed in *S. epidermidis* and may be present in other *Staphylococci*. Another form of motility has recently been observed in the *Staphylococci; “*comets”, which have characteristics of gliding motility and are similar in behaviour exhibited by known gliding bacterial species. These discoveries have the potential to have a significant impact on our understanding of *S. aureus* virulence and may offer new targets for anti-staphylococcal treatment.
